# Comparison of the Lonidamine Potentiated Effect of Nitrogen Mustard Alkylating Agents on the Systemic Treatment of DB-1 Human Melanoma Xenografts in Mice

**DOI:** 10.1371/journal.pone.0157125

**Published:** 2016-06-10

**Authors:** Kavindra Nath, David S. Nelson, Mary E. Putt, Dennis B. Leeper, Bradley Garman, Katherine L. Nathanson, Jerry D. Glickson

**Affiliations:** 1 Department of Radiology, Perelman School of Medicine, University of Pennsylvania, Philadelphia, Pennsylvania, United States of America; 2 Biostatistics & Epidemiology, Perelman School of Medicine, University of Pennsylvania, Philadelphia, Pennsylvania, United States of America; 3 Medicine, Division of Translational Medicine and Human Genetics, Perelman School of Medicine, University of Pennsylvania, Philadelphia, Pennsylvania, United States of America; 4 Abramson Cancer Center, Perelman School of Medicine, University of Pennsylvania, Philadelphia, Pennsylvania, United States of America; 5 Department of Radiation Oncology, Thomas Jefferson University, Philadelphia, Pennsylvania, United States of America; University of Alabama at Birmingham, UNITED STATES

## Abstract

Previous NMR studies demonstrated that lonidamine (LND) selectively diminishes the intracellular pH (pHi) of DB-1 melanoma and mouse xenografts of a variety of other prevalent human cancers while decreasing their bioenergetic status (tumor βNTP/Pi ratio) and enhancing the activities of melphalan and doxorubicin in these cancer models. Since melphalan and doxorubicin are highly toxic agents, we have examined three other nitrogen (N)-mustards, chlorambucil, cyclophosphamide and bendamustine, to determine if they exhibit similar potentiation by LND. As single agents LND, melphalan and these N-mustards exhibited the following activities in DB-1 melanoma xenografts; LND: 100% tumor surviving fraction (SF); chlorambucil: 100% SF; cyclophosphamide: 100% SF; bendamustine: 79% SF; melphalan: 41% SF. When combined with LND administered 40 min prior to administration of the N-mustard (to maximize intracellular acidification) the following responses were obtained; chlorambucil: 62% SF; cyclophosphamide: 42% SF; bendamustine: 36% SF; melphalan: 10% SF. The effect of LND on the activities of these N-mustards is generally attributed to acid stabilization of the aziridinium active intermediate, acid inhibition of glutathione-S-transferase, which acts as a scavenger of aziridinium, and acid inhibition of DNA repair by O^6^-alkyltransferase. Depletion of ATP by LND may also decrease multidrug resistance and increase tumor response. At similar maximum tolerated doses, our data indicate that melphalan is the most effective N-mustard in combination with LND when treating DB-1 melanoma in mice, but the choice of N-mustard for coadministration with LND will also depend on the relative toxicities of these agents, and remains to be determined.

## Introduction

Melanoma, the most deadly of all skin cancers [1], is primarily treated by surgical excision, which is curative in about 80% of patients if the tumor is detected in its early stages. However, with recurrence and distant metastases, the prognoses is very poor since effective methods for treating systemic disease are still under development and are among the most active fields of current pharmacological research. About 40–60% of melanoma patients exhibit a BRAF mutation occurring with a ninety percent frequency substitution of glutamate for valine at amino acid 600 (i.e., V600E mutation) [2]. Agents targeting this mutation exhibited considerable initial success, but response was variable and of limited duration [2]. An effort to deal with melanoma resistance to these agents has utilized MEK, RAS and other signal-transduction inhibitors used in combination [3]. Immunotherapy with Ipilumimab [4] as well as PD-1, PD-L1 checkpoint inhibitor therapy [5] is currently under clinical evaluation. Adoptive cell transfer therapy and vaccine development are also under development with some anecdotal success, but a consistent result of these approaches with any solid tumors is yet to be achieved [6, 7]. The role of melanin in therapeutic outcome of melanomas has also been studied [8–12]. As promising as these novel targeted therapies are, a curative treatment of systemic melanoma remains elusive. The most promising approach for systemic treatment of this disease will probably be the development of multiple therapeutic approaches based on different mechanisms of action that could be administered simultaneously or sequentially in order to overcome the inherent heterogeneity of melanoma and its ability to resist almost any agent based on a single mechanism of action. Towards this end, we have been exploring the use of an existing drug, LND, to selectively sensitize melanoma and other tumors to treatment with nitrogen (N)-mustards and anthracyclines [13–15]. Conventional cytotoxic chemotherapy with refined methods to increase melanoma-targeted specificity, drug delivery and minimize systemic toxicity will probably play a crucial role in a broadly based approach to the treatment of this disease. Melphalan is currently used in hyperthermic isolated limb perfusion for treatment of melanoma in transit or soft-tissue sarcomas of the limbs, and is also used in the treatment of multiple myeloma, whereas doxorubicin remains in prominent use in the treatment of a wide range of malignancies. Therefore, increasing the activities of these agents by coadministration with LND may have considerable impact on treatment of neoplastic disease. While we have previously demonstrated that LND potentiates melphalan response, we now compare this N-mustard with three additional N-mustards to determine the relative efficacy of the LND-N-mustard combination for treating melanoma.

As a consequence of high levels of aerobic glycolysis [16], tumors generally exhibit a slightly acidic extracellular pH (pHe) and a neutral to alkaline intracellular pH (pHi) leading to a plasmalemmal pH gradient, that is slightly acidic on the outside and neutral to slightly alkaline on the inside. Since normal cells do not grow well in an acidic environment, this gradient enables tumor cells to successfully compete with stromal cells during invasive tumor growth [17]. Manipulation of pHe and/or pHi of tumors impacts tumor growth, metastasis and response to therapy [18, 19]. The microenvironment of tumors can be modified by administering sodium bicarbonate in order to increase the pHe and thereby reduce tumor invasiveness [17, 20–25]. In contrast, our aim was to decrease the pHi in order to increase the intracellular activity of N-mustards. We accomplished this by administering lonidamine (LND, 100 mg/kg, intraperitoneally; i.p.), an inhibitor, of the monocarboxylate transporter (MCT) that blocks cellular export of lactic acid and also impedes mitochondrial metabolism [13–15], which would otherwise prevent accumulation of lactate in the cytosol. Phosphorus-31 magnetic resonance spectroscopy (MRS) measurements indicate that LND decreases the pHi of the tumor to a minimum of 6.33 ± 0.10 in about 80 min following i.p. administration; these lower levels of pHi are sustained for at least 3hr, whereas the bioenergetic status of the tumor (βNTP/Pi) decreases monotonically after LND treatment, falling by over 66.8 ± 5.7% in 3 hr while having no effect on pHi or bioenergetic status of muscle or brain, and only a small transient effect on pHi and bioenergetic status of liver [15]. Since acidification has been reported to enhance the activity of N-mustards [26–28], we have evaluated the effect of LND-induced acidification on three representative N-mustards, chlorambucil, cyclophosphamide, and bendamustine. These findings point to the potential utility of N-mustards when administered after LND in the systemic treatment of disseminated melanoma and perhaps also other malignancies.

## Materials and Methods

### Materials

LND was purchased from Santa Cruz Biotechnology, Inc. (Santa Cruz CA, USA). The drug (LND; 5 mg) was dissolved in 227 μL of tris/glycine buffer (22.0 mg/mL), vortexed until the solution was clear, and administered i.p. at a dose of 100 mg/kg. The buffer consisted of trizma base (1.2 g) and glycine (5.76 g) in 100 mL sterile water (final pH = 8.3). Chlorambucil was purchased from Sigma Aldrich (St. Louis, MO, USA) and was dissolved by solubilization in 70% acid ethanol followed by 10-fold dilution with PBS immediately prior to i.v. administration (20 mg/kg). Cyclophosphamide was purchased from Cayman Chemical Company, Inc. (MI, USA), dissolved in PBS and administered i.v. (40 mg/kg). Bendamustine was purchased from TCI (Tokyo Chemical Industry Co., Ltd., Tokyo, Japan), dissolved in PBS and administered i.v. (25 mg/kg).

### Human Melanoma Xenografts in Nude Mice

Male athymic nude mice (01B74) 4–6 weeks of age obtained from the National Cancer Institute, (Frederick, MD, USA) were housed in microisolator cages with access to water and autoclaved mouse chow *ad libitum*. DB-1 melanoma cells were early passage human melanoma cells derived from a lymph node biopsy of a human patient with metastatic melanoma that was excised before administration of any treatment by Dr. David Berd (Thomas Jefferson University Hospital, Philadelphia, PA). The tissue harvest protocol was reviewed and approved by the institutional review board at Thomas Jefferson University Hospital in order to protect the rights and anonymity of the patient. At that time, the committee concluded that this study was exempt from informed consent due to the samples being collected absent of patient identifiers. A melanoma lung metastasis from a previously untreated patient was excised for therapeutic purposes associated with immunotherapy in 1989. The explant was cooled on ice immediately after surgery, as described by Hill LL et al. [29]. A portion of the excised tissue was disaggregated to a single cell suspension by collagenase and DNAase. The presence of melanoma cells was verified by melanoma antigens [30]. Cell suspensions were then frozen in 10% DMSO and then stored in liquid nitrogen. For experiments, cell suspensions were rapidly thawed, counted in an electronic counter (Coulter Diagnostics, Hialeah, FL) and kept on ice in aliquots of 25x10^6^ cells in 2 ml before inoculation subcutaneously for growth as xenografts. The DB-1 line was serially transplanted twice as subcutaneous xenografts in SCID mice. After the second transfer, cells were disaggregated and grown in monolayer culture at 37°C and pHe 7.3 in 5% CO_2_ in α-MEM supplemented with 10 FBS, 12 mM glucose, 10 ml/L nonessential amino acids solution and 2.9 g/L glutamine. Culture medium contained penicillin-streptomycin antibiotics for the first three weeks, and then cells were cultured in antibiotic free medium. Cells were maintained in exponential growth and passaged twice a week for 16 passages. The doubling time of the cells was approximately 36 hrs, and the plating efficiency (PE) was approximately 60%. These cyro-preserved cells were the source of inoculum used for our experiments. Cultures were not propagated longer than 2 months before being discarded and replaced by new outgrowth of frozen cells. Periodically, frozen cell stocks had to be replenished by another 16^th^ passage amplification. Cells were monitored periodically to be free of mycoplasma contamination. Over the years using this process, the cell cycle time, PE and morphology were unchanged. During this period, the doubling time for DB-1 xenografts has remained unchanged at about 5 days. DB-1 melanoma cell preparation and inoculation in male athymic nude mice and tumor volume measurement were performed as described previously [15].

### Chemotherapy with Chlorambucil, Cyclophosphamide and Bendamustine and in DB-1 Human Melanoma Xenografts

When tumors of human melanoma xenografts (DB-1) reached 20.2 ± 3.0 mm^3^, 8 cohorts of age- and weight-matched animals were randomized to the following treatment groups: Cohort 1 (sham-treated control) administered sham i.p. injections of tris/glycine buffer and was infused intravenously (i.v.) with PBS; Cohort 2 was infused i.v. with PBS 40 min after LND administration i.p. (100 mg/kg); Cohort 3 was injected i.p. with tris/glycine buffer and infused i.v. with chlorambucil (20 mg/kg); Cohort 4 was injected i.p. with tris/glycine buffer and infused i.v. with cyclophosphamide (40 mg/kg). Cohort 5 was injected i.p. with tris/glycine buffer and infused i.v. with bendamustine (25 mg/kg). Cohort 6 was infused i.v. with chlorambucil (20 mg/kg) 40 min after LND administration i.p. (100 mg/kg). Cohort 7 was infused i.v. with cyclophosphamide (40 mg/kg) 40 min after LND administration i.p. (100 mg/kg). Cohort 8 was infused i.v. with bendamustine (25 mg/kg) 40 min after LND administration i.p. (100 mg/kg). Values shown are means ± SEM; n = 5 animals for controls, LND, chlorambucil, cyclophosphamide, bendamustine, LND + chlorambucil, LND + cyclophosphamide, and LND + bendamustine treated animals.

During the treatment and sham-treatment procedures, all animals were anesthetized with ketamine hydrochloride (10 mg/mL) and acepromazine (1 mg/mL) with additional anesthesia being re-administered approximately every 45–60 min to maintain sedation. Animals were placed on a water pad heater (Gaymar T-Pump, Gaymar Industries, Inc., Orchard Park, NJ, USA) to maintain body temperature during anesthesia. Tumor dimensions were measured as well as body weight. To prevent blood clotting, tail vein catheters (I.V. Catheters FEP, Tyco Healthcare) filled with heparin (100 USP Units/mL) were placed using a restrainer (MTI Braintree Scientific, Braintree Scientific Inc., Braintree, MA, USA). After treatment, catheters were removed and animals were allowed to recover in cages. For the first five days post-treatment, tumor volume and animal weight were measured daily with calipers (Scienceware, Bel-Art Products, Wayne, NJ, USA) and scale (Acculab PP401, H & C Weighing Systems, Columbia, MD, USA), respectively. Afterwards, these measurements were repeated every other day. The tumor dimensions were measured with a caliper in three orthogonal directions, and the volume was calculated using the equation, *V* = *π*(*a* × *b* × *c*)/6, where *a*, *b*, and *c* are the length, width, and depth of the tumor. All animal studies were performed in accordance with a protocol approved by the University of Pennsylvania Institutional Animal Care and Use Committee (IACUC).

### DNA Purification, Library Preparation, and Sequencing

DNA purification was performed using the DNeasy Blood & Tissue Kit (Qiagen). Five hundred ng of genomic DNA was sheared randomly into 200 bp fragments with the Covaris^TM^ S200 UltraSonicator (Covaris®). Sheared DNA was A-tailed and ligated with adaptor-embedded indices using the NEBNext^®^ Ultra^TM^ DNA Library Prep Kit for Illumina^®^ (New England BioLabs, Inc.). DNA quality, fragment size, and concentration of library preps were measured using Agilent’s DNA 1000 chips in conjunction with the 2100 Bioanalyzer (Agilent Technologies). Samples were equimolarly pooled prior to capture with a 2.2 Mbp SureSelect^XT^ Custom Target Enrichment Kit (Agilent Technologies) targeting 108 genes previously implicated in melanomagenesis. Paired-end sequencing was performed on the HiSeq^TM^ 2000 sequencing system (Illumina) at the Perelman School of Medicine Next-Generation Sequencing Core Facility.

### Mutational Analysis

Short-sequenced reads were aligned to the hg19 human reference genome using the Burrows-Wheeler Alignment (BWA) tool [31]. Duplicate reads were removed, as well as reads that map to more than one location, off-target reads, and variants annotated with the incorrect transcript. The Genome Analysis Toolkit (GATK) was used for data quality assurance as well as for Single Nucleotide Variant (SNV) and small insertion and deletion (indel) calling [32, 33]. After down-sampling by GATK, a mean target coverage of 197X was achieved. Variants were annotated with wANNOVAR [34].

### Copy Number Variation Prediction

Prediction of copy number variation from sequencing data was performed using CODEX [35]. This algorithm normalizes the data using a Poisson latent factor model that removes biases due to GC content, exon capture, amplification efficiency, and latent systemic artifacts. Six latent factors were used for the normalization of the dataset in this study. Segmentation was restricted to exons only for all genes. Only homozygous loss and high amplification calls are reported. Log_2_ ratio thresholds used for high amplification and homozygous loss were 1.33 (copy number five) and -1.2, respectively. Visual confirmation of SNV calls was accomplished with Nexus 7.5 (BioDiscovery, Inc.) software.

### Statistical Analyses

The approach to the primary analysis followed methods described by Demidenko [36]. The analysis plan was designed to address the question of whether each alkylating agent delays tumor growth, and whether the addition of LND further delays tumor growth relative to the alkylating agent alone. The data were first visualized using individual growth curves and smoothed using locally weighted scatterplot smoothing (loess). For each animal, we determined the time from treatment until the tumor reached a volume of 150 mm^3^, or approximately 3 doublings from its initial volume at the time treatment began. The treatment delay was estimated from the difference in the mean time to 150 mm^3^ between the (T) treated and (C) control arm and p-values determined using a Wald test. To determine the proportion of tumor cells surviving, we first computed the slope of the log tumor volume curve for each animal. This slope was computed using pre-treatment data for the treated animals and both the pre-treatment data and the data up to day 20 post-treatment in the controls, where the log tumor volume curve for the controls remained linear. We then computed the mean slope of the growth curves (*B*) across both arms of the study and estimated the proportion of the tumor cells surviving treatment as *exp*(−(*T*−*C*) × *B*). This estimate was truncated at a maximum of 1.0 as surviving cells cannot exceed 100%. Standard errors of the estimates were computed using the bootstrap percentile method and used to construct Wald tests. We further compared the data from these experiments to our previous work on melphalan in the absence of glucose [14]. While these data were analyzed using the same methods described here, the initial tumor volume in the earlier experiment was larger; thus, the target volume was four times the initial volume, and the control data were terminated at day 6 post-treatment. We used one-sided tests and a type I error rate of 0.05. To be consistent with the one-sided p-value, we present two-sided 90% confidence intervals obtained by the bootstrap percentile method.

We used weight loss as a measure of toxicity. The minimum weight for each animal in the first 21 days post-treatment was normalized to its median pre-treatment weight. The log of this ratio was the outcome in a series of linear models where the predictor was the alkylating agent with or without LND. P-values were one-sided as it is of scientific interest to determine whether the alkylating agents increased weight loss relative to the control, and whether the combination caused increased weight loss relative to the individual agent. Analyses were conducted in R Studio (V 99.484) using R (Version 3.2.1; R Foundation for Statistical Computing, Vienna, Austria) [37].

## Results

**Fig 1:**
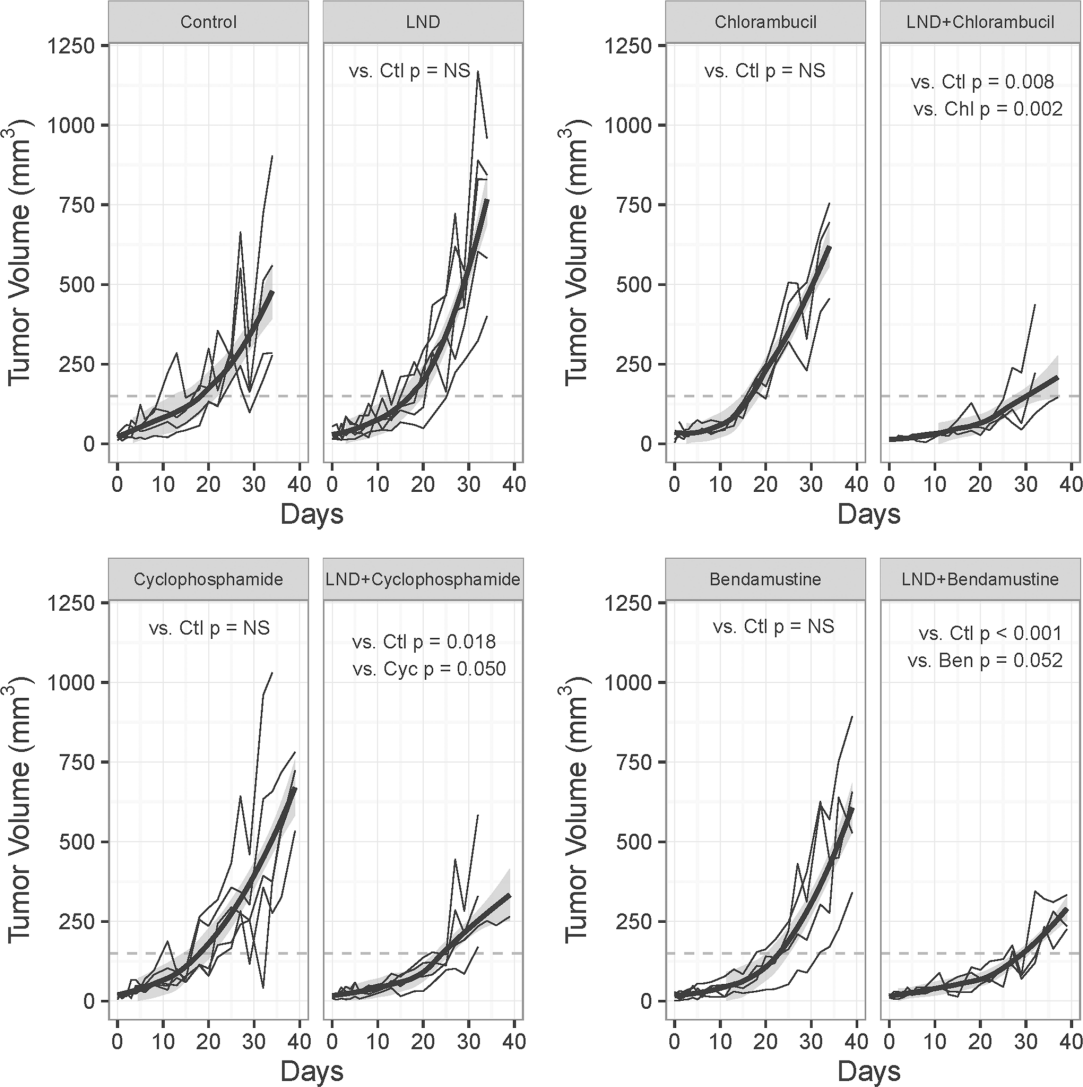
Growth Delay Experiments on DB-1 Melanoma Xenografts. Growth delay experiments performed on DB-1 human melanoma xenografts in nude mice treated with 20 mg/kg chlorambucil, 40 mg/kg cyclophosphamide or 25 mg/kg bendamustine. Mice were treated on Day 0 as follows: control (sham i.p. tris/glycine buffer + sham i.v. PBS), LND (lonidamine; 100 mg/kg; i.p.), chlorambucil, LND + chlorambucil, cyclophosphamide and LND + cyclophosphamide, or bendamustine, LND + bendamustine. Each grey line is the trajectory for an individual animal. The trend for each group is illustrated using the loess smoothed curve (dark line) and its standard error (shaded area). The time at which each animal’s tumor achieved a volume of 150 mm^3^ (dashed line) was used to estimate the mean tumor growth delay (T-C) shown in Table 1. P-values for the difference in mean growth delay for each pair of plots are shown. Values labeled NS did not achieve the threshold of 0.05. P-values for the mean difference in cell survival rates appear in Table 1. Abbreviations are as follows: Ctl, Control; Chl, Chlorambucil; Cyc, Cyclophosphamide; Ben, Bendamustine; NS, Not Significant.

The effects of treatment with LND in combination with chlorambucil, cyclophosphamide and bendamustine (Fig 1) were evaluated using tumor growth delay experiments. For these experiments, mean tumor-doubling times ranged from 4.6 days (LND alone) to 11.0 days (bendamustine alone). These rates are comparable to those observed in our earlier experiences with melphalan (doubling times of 5.3 to 5.9 days).

Compared to control (Cohort 1), Table 1 shows that LND alone neither significantly delayed tumor growth nor altered cell survival. Similarly, each of the alkylating agents, when administered as single agents (Cohorts 3–5), yielded no statistically significant differences relative to control for either mean tumor growth delay or cell surviving fraction (Table 1). Among the three alkylating agents administered individually, the largest mean growth delay of 3.8 days occurred for bendamustine (p = 0.11) which also exhibited the most reduced cell surviving fraction of 79% (p = 0.28).

**Table 1:** Summary of estimated mean growth delay (T-C), and surviving fraction as a percent (100×exp(−(T−C)×B), with bootstrap 95% CI), log cell kill method, by experiment and treatment arm in DB-1 human melanoma xenografts. Individual growth curve data appear in Fig 1.

Experimental Groups	Growth Delay (T-C; days)			Surviving fraction (%) Estimates (90% CI)		
	Estimate (90% CI)	p value		Estimates (90% CI)	p value	
		Vs Control	Vs Single Agent		Vs Control	Vs Single Agent
**Lonidamine (LND)**	-2.1*	0.73	NA	100	0.67	NA
	(-7.4, 2.9)			(59, 100)		
**Chlorambucil**	-4.1*	0.96	NA	100	0.81	NA
	(-8.1, 0.2)			(98, 100)		
**Cyclophosphamide**	-1.3	0.65	NA	100	0.63	NA
	(-6.1, 3.6)			(62, 100)		
**Bendamustine**	3.8	0.11	NA	79	0.28	NA
	(-1.8, 9.8)			(42, 100)		
**Melphalan**	7.6	0.007	NA	41	< 0.001	NA
	(2.3, 14.6)			(16, 75)		
**LND + Chlorambucil**	7.7	0.008	0.002	62	0.004	0.090
	(2.5, 13.1)			(35, 86)		
**LND + Cyclophosphamide**	5.9	0.018	0.050	42	0.001	0.068
	(0.6, 10.8)			(19, 92)		
**LND + Bendamustine**	10.6	<0.001	0.052	36	< 0.001	0.13
	(4.9, 16.0)			(20, 62)		
**LND + Melphalan**	17.8	<0.001	0.015	10	< 0.001	0.247
	(10.8, 23.8)			(5, 24)		

**Note:** Mean growth delay and survival estimates for LND (lonidamine) alone, and for each of the three alkylating agents, either alone or in combination with LND. All p values are one-sided. Doubling times are assumed identical across all treatments. Compared to control, LND alone did not yield significant differences in either growth delay (p = 0.73) or percent survival (p = 0.67). NA is not applicable.

*Negative growth delay indicates return to tumor volume at an estimated rate that was slightly faster than the control (Fig 1). The 90% CI in all cases where the estimate was negative covered zero, suggesting that these differences represent random variation among animals.

In sharp contrast, each of the alkylating agents in combination with LND (Cohorts 6–8) showed both statistically significant growth delays and statistically significant reductions in cell surviving fraction relative to the control group (Cohort 1). Specifically, compared to control, the mean growth delay for each of the combinations of LND with an alkylating agent ranged from 5.9 to 10.6 day (p < 0.05 for each agent in combination with LND, see Table 1 or Fig 1 for specific individual p-values). Similarly, compared to control, the mean surviving fraction for each of the combinations ranged from 41 to 62% (p < 0.01 for each agent in combination with LND, see Table 1 for specific p-values).

We next compared the response to the combination of LND plus an alkylating agent to the alkylating agent alone. Here we found that the mean growth delay for each of the alkylating agents, combined with LND, yielded a mean growth delay that achieved or approached statistical significance. Specifically the mean growth delay for LND + chlorambucil (Cohort 6) exceeded chlorambucil alone Cohort 3 (p = 0.002) and the mean growth delay for LND + cyclophosphamide (Cohort 7) exceeded cyclophosphamide alone (p = 0.050). The mean growth delay for LND + bendamustine (10.6 days, Cohort 8) exceeded bendamustine alone (3.8 days, Cohort 5,) but this result did not achieve strict statistical significance (p = 0.052) (Table 1).

For each of the three alkylating agents, the estimate of the cell surviving fraction for the combination with LND was smaller than for the alkylating agent alone. However, in no case did the differences achieve strict statistical significance. We note that the surviving cell fraction for LND + cyclophosphamide was 42% versus 100% for cyclophosphamide alone, and that the surviving cell fraction for LND + chlorambucil was 62% versus 100% for chlorambucil alone, with p < 0.10 for both contrasts (Table 1).

In previous work we showed that the mean growth delay for LND + melphalan (17.8 days, previously published results) exceeded melphalan alone (7.6 days, previously published results, p = 0.015). The mean difference in cell surviving fraction for LND + melphalan (10%, previously published results) versus melphalan alone (41%, previously published results) did not achieve significance [14, 15].

LND + chlorambucil (Cohort 6), LND + cyclophosphamide (Cohort 7) and LND + bendamustine (Cohort 8) resulted in a 38%, 58% and 64% cell kill, respectively. In our previous study LND + melphalan yield 90% cell kill, respectively, [14, 15] (Table 1).

### Weight Loss

**Fig 2:**
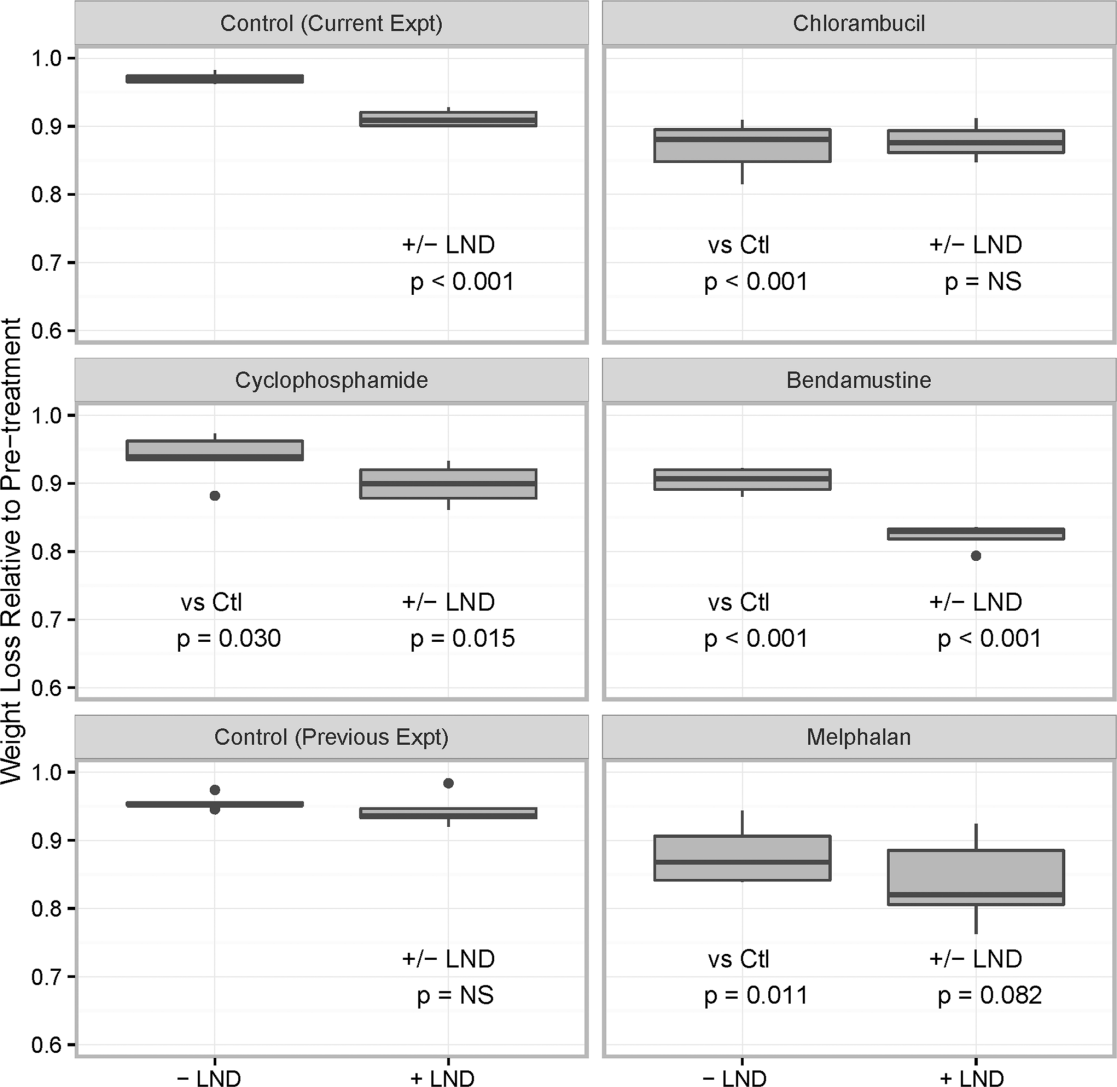
Weight Loss Experiments on DB-1 Melanoma Xenografts. Box plot of weight loss relative to median pretreatment weight for the control and each alkylating agent with (+) or without (-) LND (lonidamine). Bold horizontal lines indicate median, boxes indicate interquartile range, (IQR) whiskers extend to the smaller of either the range of the data or the box +/- 1.5 IQR. Outliers appear as dots. Abbreviations are as follows: Ctl, Control; NS, Not Significant.

For each cohort, Fig 2 displays boxplots of the maximum weight loss, relative to the pre-treatment value. The mean pre-treatment weight across all animals was 30.1 g (95% CI of 29.3, 30.3). Without LND, the average weight of the controls post- versus pre-treatment was 97.0% (90% CI 96.1, 97.9%, p < 0.001). With LND, the average weights of the controls post- versus pre-treatment was 91% (90% CI 90.2, 92.0) of control, and significantly lower than in the absence of LND (p < 0.001). Fig 2 and Table 2 show that for the alkylating agents alone, the mean post-treatment weight declined to levels that significantly exceeded that of the weight loss observed in the control without LND. The pre-treatment: post-treatment weight reduction ranged from 89.4% (chlorambucil, Cohort 3, p < 0.001 versus control without LND) to 96.6% (cyclophosphamide, Cohort 4, p = 0.030 vs control without LND) (Table 2).

**Table 2:** Weight loss of DB-1 human melanoma xenografts following treatment with nitrogen-mustards alone or in combination with LND

Experimental Groups	Ratio of Post-treatment: Pretreatment Weight (% Relative to Control)		
	Estimate (90% CI)	p value	
		Vs Control	Vs Single Agent
**Alkylating Agent Alone**			
**Chlorambucil**	89.4	< 0.001	NA
	(86.1, 92.8)		
**Cyclophosphamide**	96.6	0.030	NA
	(93.8, 99.5)		
**Bendamustine**	93.2	< 0.001	NA
	(91.3, 95.1)		
Melphalan*	92.0	0.011	NA
	(86.8, 97.4)		
**Alkylating Agent in Combination with LND**			
**LND + Chlorambucil**	90.5	< 0.001	0.627
	(87.1, 93.9)		
**LND + Cyclophosphamide**	92.6	0.001	0.015
	(89.7, 95.5)		
**LND+ Bendamustine**	84.7	< 0.001	< 0.001
	(83.0, 86.4)		
LND + Melphalan*	87.7	0.001	0.082
	(83.0, 92.6)		

**Note**: Ratio of minimum post-treatment to pre-treatment weight for each group, normalized to the ratio in the control group. P-values are for the comparison to control, and for the comparison of the combined LND (lonidamine)-alkylating agent to the alkylating agent alone. The mean weight at the start of treatment for animals in the current experiment was 30.1 g (95% CI 29.3, 30.3). For the control group the relative post- to pre-treatment weight was 97.0% (90% CI of 96.1, 97.9%).

* Melphalan data was adopted from previous publication [14, 15].

For the animals treated with the alkylating agent in combination with LND, post-treatment relative to pre-treatment weight ranged from 84.7% (LND + bendamustine, Cohort 8) to 92.6% (LND + cyclophosphamide, Cohort 7) (Table 2). For chlorambucil, the weight loss was similar with or without LND. In contrast, for cyclophosphamide and bendamustine, the estimated weight loss was significantly lower than for the alkylating agent without LND (p < 0.015 and p < 0.001 respectively) (Table 2).

We note that, the minimum post versus pre-treatment weight of animals receiving melphalan (92%, p = 0.011) or LND + melphalan (87.7%, p = 0.001) was significantly lower than control, but that differences between the two groups were not significant (p = 0.082) [15] (Table 2).

**Fig 3:**
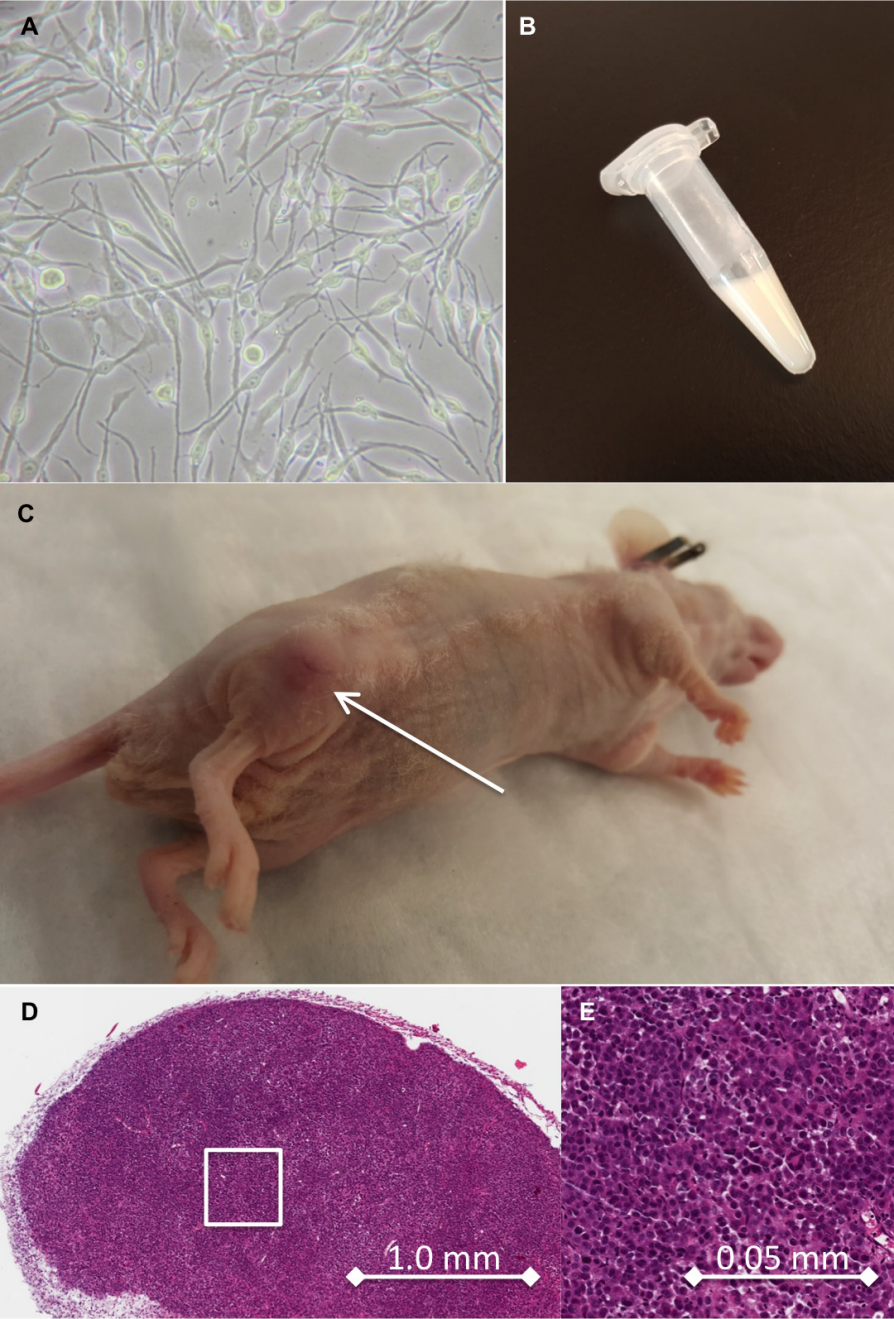
*In vitro*, *In vivo* and *Ex vivo* Characteristics of Human DB-1 Melanoma Cells. (A) DB-1 cells in culture at 40X magnification. (B) Cell pellet demonstrating amelanotic characteristic of cells. (C) Typical xenograft tumor on flank of an athymic nude mouse. Arrow pointing towards lesion. (D) H&E stain at 42X magnification of section through harvested tumor. Inset represents region shown in panel E at 840X magnification.

Fig 3 shows *in vitro*, *in vivo* and *ex vivo* characteristics of human DB-1 melanoma cells. S1 Spreadsheet shows tumor growth delay and body weight data.

### Mutations and Copy Number Aberrations in the DB-1 Human Melanoma Cell Line

The DB-1 cell line contained the following known pathogenic mutations: *BRAF* p. V600E; *TP53* p.L145R; *CDK4* p.R24C and *TERT* promoter region Chr5:1295228–1295229 GG>AA. Additional variants identified were *PTEN* p.T176A, *JAK3* p.P693L, *EGFR* p.P753S, and *APC* p.140G. The cell line also contained homozygous loss of *CDKN2B*.

## Discussion

The Warburg effect has been exploited for the detection and treatment of cancer for many years. PET (Positron Emission Tomography) imaging with FDG (fluorodeoxyglucose) has utilized it for the minimally invasive and sensitive detection of cancer. We reasoned that if we could trap the lactate produced by tumor cells inside the cytosol, we would have a method to selectively acidify cancer cells and make them susceptible to alkylating agents whose activity is enhanced by acid [13–15, 38] and whose lethal effect is also enhanced under acidic conditions. The goal of the present study was to determine whether intracellular tumor acidification and deenergization could increase the efficacy of bendamustine, cyclophosphamide and chlorambucil in DB-1 melanoma xenografts following LND administration. Previous studies had indicated that i.p. injection of LND at 100 mg/kg produced the optimal effects on tumor acidification and bioenergetic decline without significant toxicity to normal tissues. N-mustards are generally administered by i.v. infusion, and were administered at levels comparable to those used by other investigators in studies of mouse xenografts [15]. Compared to the alkylating agent alone, we observed significant, or near-significant increases in growth delays for all three alkylating agents when combined with LND. Additionally, compared to control each alkylating agent in combination with LND showed statistically significant cell kill, with rates ranging from 38 (Chlorambucil) to 64% (Bendamustine). In contrast, the alkylating agents alone demonstrated very little cell kill with rates ranging from 0 to 21%. We note that cell kill rates for the combination of alkylating agent plus LND compared to the alkylating agent alone did not achieve strict statistical significance (p values ranging from 0.068 to 0.13). We chose to report the experiment as planned, rather than repeating it, given the statistical comparison to the control, and the consistent pattern in both growth delay and cell kill across all three agents in combination with LND. In our experiment relative cytotoxicities of these agents in DB-1 melanoma xenografts after LND administration were bendamustine (cell kill, 64%) > cyclophosphamide (cell kill, 58%) > chlorambucil (cell kill, 38%). These differences in cytotoxicities are small and conclusions regarding the relative toxicity of these agents would require a substantially larger number of animals than was used in the current study. We have previously reported cytotoxicity of melphalan (cell kill, 90%) following LND administration in DB-1 melanoma xenograft [14], suggesting that among the N-mustards, melphalan may exhibit the highest level of potentiation following LND treatment. This conclusion must be taken with some caution as (1) confidence intervals were broadly overlapping and (2) the experiments were not conducted concurrently, and (3) we cannot strictly rule out differences between experimental conditions for the two sets of results. Lastly, we note that the three alkylating agents examined here were associated with a 3.4% to 10.6% increase in post-treatment weight loss relative to control, while the combination of agents with LND were associated with a 7.4% to 15.3% increased weight loss relative to control, values that for cyclophosphamide and bendamustine were significantly larger than for the alkylating agent alone. The effect on weight loss is similar to those observed for melphalan.

In preparation for studies combining LND with alkylating agents, we have already performed *ex vivo* investigations of the effects of a number of candidate platinum and alkylating agents on cultured DB-1 melanoma cells [15]. Melphalan exhibited the greatest cytotoxicity as a single agent, followed by cisplatin, bendamustine and chlorambucil. LND also exhibited a low level of cytotoxicity as a single agent in these experiments. This most likely occurred due to the presence of serum in the medium, thus limiting the bioavailability of LND to exhibit an effect. Cyclophosphamide was not included in *ex vivo* study at that time. Recently, we have demonstrated by ^31^P MRS (Phosphorus Magnetic Resonance Spectroscopy) that the treatment of DB-1 melanoma xenografts with LND (100 mg/kg, i.p.) selectively acidifies and de-energizes tumors [15].

The activity of N-mustards increases with increasing acidification of tumors [26–28, 39–42]. In the case of N-mustards, this could be caused by three effects: (i) increased concentrations of the active intermediate cyclic aziridinium ion; (ii) decreased concentrations of competing nucleophiles, such as hydroxide and glutathione, whose production is diminished by decreased activity of gluthathione-S-transferase under acidic conditions; and (iii) decreased DNA repair as a result of acid inhibition of O^6^-alkyltransferase [40, 42]. This is probably largely because acid shifts the equilibrium between the various forms of these agents towards more active forms. In the case of N-mustards, the active species is the cyclic aziridinium ion. In cells, the reactivity of these agents will also be affected by considerations of transport into the cell and, eventually, into the nucleus, where DNA alkylation occurs. Elimination of these agents by active transport via multidrug resistant pumps will also modify the activities of melphalan [43] and other N-mustards. Since multidrug resistance is an energy consuming process, deenergization of the tumor should increase the retention and, hence, also the activities of these alkylating agents in tumor cells.

Differences were observed between the three groups but did not achieve statistical significance. This may reflect the sample size within each group, which was chosen with the primary goal of determining differences between the individual agent and the combination with LND. With this limitation in mind, it is of interest that bendamustine exhibited the longest growth delay and largest cell kill. Previous reports indicated that increased efficacy of bendamustine might be due to inhibition of mitotic checkpoints, inefficient DNA repair, and initiation of p53-dependent DNA-damage stress response, all of which lead to mitotic catastrophe and apoptosis [44]. Moreover, the presence of a benzimidazole ring in addition to the N-mustard group may influence the way bendamustine interacts with DNA and/or its antimetabolite properties [44]. The benzimidazole central ring system is unique to bendamustine; the intent of adding this structure to the N-mustard was to include the antimetabolite properties of benzimidazole. This heterocyclic ring structure may contribute to the unique antitumor activity of bendamustine and distinguish it from conventional 2-chloroethylamine alkylators-cyclophosphamide, chlorambucil and melphalan [44].

Studies have shown that melanotic melanomas exhibited significantly shorter disease-free survival and overall survival than those with amelanotic lesions. Similarly, melanin-producing lymph node metastases were linked to shorter overall survival and disease-free survival, which was confirmed by a significantly longer mean/median disease-free survival for amelanotic versus melanotic metastases [9]. Bryoanza et al. have shown that melanin content also acts as a radioprotector and scavenger resulting in changing of properties of melanoma cells [10]. Melanogenesis is a metabolic pathway characteristic of normal and malignant melanocytes that can affect the behavior of melanoma cells or their surrounding environment, and the inhibition of melanogenesis might represent a valid therapeutic target for the management of advanced melanotic melanomas [11]. However, in the current study, with the DB-1 melanoma cell pellet indicating a very low content of melanin, based on a lack of colorization (amelanotic), DB-1 is probably an ideal cell line to examine these therapeutic effects independent of pH interaction with melanin. A possible future direction would be to examine LND effects on pH in a xenograft model that is more highly expressive of melanin.

Unfortunately, we have no clinical history of the response of the primary tumor in donor from whom the DB-1 cell line originated. Nor do we have any information regarding the treatment response of the donor’s metastases. The DB-1 cell line, besides being a cancer cell model, is a model of metastatic disease. It is hoped that the results we observe with DB-1 xenografts, investigating the tumor sensitizing effects of LND, are a guide to the response to treatment with alkylating agents of patients with metastatic melanoma.

Earlier studies have supported the mechanism of MCT inhibition by LND [15, 45–47]. MCT-1 and MCT-4, the other isoform of MCT present in DB-1 melanoma [48], have K_m_ values of about 4.5 mM and 22 mM for lactate, respectively [49]. Recent LC-MS (Liquid chromatography-mass spectrometry) analysis of the effects of LND on DB-1 melanoma cells indicates that this agent also inhibits complex II at the ubiquinone reduction step [50]. Furthermore, our recent data demonstrates that LND inhibits the MPC of isolated liver and heart mitochondria with an IC_50_ of 2.5 μM [51]. Thus, the most potent site of activity of LND is the MPC followed by MCT1 and MCT4, and complex II (in that order) at increasing concentrations of LND.

Our group and other researchers have identified the sites of action of LND that are common to both normal tissue and melanoma [50, 51]. Explanation for why melanoma and other cancers demonstrate selectivity for metabolic response to LND compared to normal tissues needs further investigation. Oncogenic mutations of BRAF have been implicated in changes of the metabolic phenotypes seen in melanoma favoring a shift towards more oxidative and less glycolytic metabolism [52, 53]. Prior to treatment the DB-1 tumor derives about equal amounts of its ATP from both of these pathways [54–56]. LND should decrease mitochondrial ATP production to a greater extent than glycolytic energy production since it inhibits mitochondrial uptake and oxidation of pyruvate resulting in a decrease in TCA cycle flux, and it also inhibits electron transport, while the concentration of lactate (and, hence, the extent of glycolysis) in the tumor triples [15]. Therefore, the effect of shifting tumor metabolism in the direction of oxidative metabolism as a result of the BRAF-V600E mutation makes the tumor more responsive to LND and agents that it potentiates such as N-mustards and anthracyclines.

Limited success has been achieved in treating melanomas with agents that target the BRAF-V600E mutation observed in about 50% of melanomas [2] and with ipilumumab [57, 58], but none of these have been curative. Development of resistance following oncogene inhibition is common in the clinic, where tumor cells acquire or develop pro-survival mechanisms to maintain their growth [59, 60]. While BRAF inhibitors have displayed a rapid and dramatic response rate in BRAF mutated melanoma patients, most of these patients eventually relapse [61, 62]. Studies have shown the increased expression of genes involved in TCA cycle, oxidative phosphorylation and ATP generation in melanomas with mutant BRAF treated with vemurafenib, an inhibitor of BRAF-V600E [52]. Combination of an immunotherapeutic agent and a BRAF inhibitor may help overcome oncogene inhibition resistance [63], and, as noted above, LND, should make the mutant BRAF more responsive to N-mustards and anthracyclines. The most promising approach for the systemic treatment of this disease will probably involve the development of multiple therapeutic agents functioning by a variety of independent mechanisms that would be very difficult to simultaneously circumvent. The present study demonstrates one such method that utilizes the Warburg effect and agents that trap lactate in tumors to selectively acidify melanoma and sensitize it to systemic therapy with conventional alkylating agents.

## Supporting Information

S1 Spreadsheet Tumor growth delay and body weight data spreadsheet. (XLSX)

## Abbreviations

LND: lonidamine
MRS: magnetic resonance spectroscopy
MCT: monocarboxylate transporter
pHi: intracellular pH
pHe: extracellular pH
i.p.: intraperitoneal
i.v.: intravenous
s.c.: subcutaneous
NTP: nucleoside triphosphate
CHC: α-cyano-4-hydroxycinnamic acid
PD-1: programmed cell death-1
PD-L1: programmed cell death ligand-1
